# New Treatment Perspectives in Adolescents With Anorexia Nervosa: The Efficacy of Non-invasive Brain-Directed Treatment

**DOI:** 10.3389/fnbeh.2018.00133

**Published:** 2018-07-20

**Authors:** Floriana Costanzo, Deny Menghini, Antonella Maritato, Maria C. Castiglioni, Alberta Mereu, Cristiana Varuzza, Valeria Zanna, Stefano Vicari

**Affiliations:** Child Neuropsychiatric Unit, Department of Neuroscience, Bambino Gesù Children Hospital, Rome, Italy

**Keywords:** tDCS, anorexia, prefrontal cortex, adolescents, BMI

## Abstract

Poor treatment outcomes are available for anorexia nervosa (AN) and treatment innovations are urgently needed. Recently, non-invasive neuromodulation tools have suggested to have potential for reducing an symptomatology targeting brain alterations. The objective of the study was to verify whether left anodal/right cathodal prefrontal cortex transcranial direct current stimulation (tDCS), may aid in altering/resetting inter-hemispheric balance in patients with AN, re-establishing control over eating behaviors. Twenty-three adolescents with an underwent a treatment as usual (AU), including nutritional, pharmacological, and psychoeducational treatment, plus 18 sessions of tDCS (TDCS+AU = n11; mean age = 13.9, *SD* = 1.8 years) or a family based therapy (FBT+AU = n12, mean age = 15.1, *SD* = 1.5 years). Psychopathological scales and the body mass index (BMI) were assessed before and after treatment. After 6 weeks of treatment, the BMI values increased only in the tDCS group, even at 1-month follow-up. Independently of the treatment, all participants improved in several psychopathological measures, included AN psychopathology and mood and anxiety symptoms. Our results demonstrated for the first time a specific effect of the left anodal/right cathodal tDCS treatment protocol on stable weight gain and a superiority compared to an active control treatment for adolescents with AN. Results were interpreted as a possible direct/indirect effect of tDCS in into some pathophysiological mechanisms of AN, involving the mesocortical dopaminergic pathways and the promotion of food intake. This pilot study opens new perspectives in the treatment of an in adolescence, supporting the targeted and beneficial effects of a brain-based treatment.

## Introduction

Eating disorders (ED) are highly distinctive disorders characterized by pathological eating behaviors and body image disturbance. according to the diagnostic and statistical manual of mental disorders, fifth edition (Dsm-5), ED shall be considered as a spectrum of over-eating and under-eating, associated with altered weight and with altered food reward that results in significant biologic, psychological, and social complications. Anorexia nervosa (AN) is a severe ED associated whit other important physical and psychological comorbidities. It is defined by an extremely low Body Mass Index (BMI < 18.5 kg/m^2^) and concomitant anxiety and preoccupations related to weight and body image, and exerts a significant individual and societal impact. Levels of mortality and disability and lifetime prevalence in AN are high (Arcelus et al., 2011; Smink et al., 2012; Walsh, 2013). Onset is increasing in the early adolescence, mainly in the females, with a symptomatology most common in teen-ager and adults.

To data, available treatments for AN are only moderately effective. Pharmacological treatment plays a limited role (Aigner et al., 2011; Gustavsson et al., 2011; Dold et al., 2015; Garner et al., 2016). Selective serotonin reuptake inhibitors (SSRIs) and neuroleptics constitute the mainstay treatments, but the efficacy of these medications is fairly poor and up to 30% of patients with AN prove to be medically intractable (Steinhausen, 2002; Kontis and Theochari, 2012). In turn, no Food and Drug Administration indication for any pharmacological treatment for AN is given.

Psychological therapies, such as Cognitive Behavioral Therapy and Family Psychotherapy, are widely considered the treatment of choice. However, no single psychological intervention has shown clear superiority in treating adults with AN, while in adolescents with AN, the evidence base is strongest for the use of Family Psychotherapy over alternative individual psychotherapies (Lock et al., 2010; Herpertz-Dahlmann and de Zwaan, 2011; Le Grange et al., 2012; Agras et al., 2014; Rienecke, 2017). Recently, it was shown the efficacy of integrated treatment as opposed to single therapies, as suggested by the current guidelines for the treatment of ED in children and adolescents (National Institute for Clinical Excellence, 2004; American Psychiatric Association, 2006). In particular, the application of a Multifocal Integrated Treatment, based on both family and individualized psychotherapy, nutritional and pharmacological interventions, showed improvement in the eating psychopathology in adolescents with AN (Laghi et al., 2017). However, there is a need for continued efforts to develop novel interventions (Bodell and Keel, 2010), since evidence-base treatment for AN is still lacking.

Novel approaches to treatment of ED in general and specifically for AN have been called for by august bodies such as the National Institute for Health and Care Excellence (NICE) in the UK and the National Institutes of Health (NIH) in the USA. Recently, neuromodulation procedures, which are emerging techniques that can be used to stimulate or inhibit neural activity, has been postulated as a potential treatment for ED (for recent review see McClelland et al., 2013; Lee et al., 2018).

Marked increase in neuromodulation approaches for treatment of ED grounds on recent year's extensive neuroscience data related to ED and to the emergence of testable neurobiological models. Altered activity in the insula (Kaye et al., 2010; Mohr et al., 2010; Bär et al., 2013; Oberndorfer et al., 2013; Strigo et al., 2013), abnormalities in the processing of rewards (Bohon and Stice, 2011; Avena and Bocarsly, 2012; Ritschel et al., 2017) and alterations in frontal regions have been reported (Brooks et al., 2011; Celone et al., 2011; Strigo et al., 2013; Kullmann et al., 2014). Subsequently, neural models of ED have been developed (Steinglass and Walsh, 2006; Brooks et al., 2011; Friederich and Herzog, 2011; Riva, 2016; Steinglass et al., 2017).

In AN, some studies considered abnormalities in dorsolateral prefrontal cortex (DLPFC) (Kaye et al., 2009; Van Kuyck et al., 2009; Brooks et al., 2011; Ehrlich et al., 2015; Hestad et al., 2016). Furthermore, fMRI studies, EEG measurements and PET scan in individuals with AN showed a hyperactivity of right hemisphere (Grunwald et al., 2004; Brooks et al., 2011; Bär et al., 2013; Phillipou et al., 2015) and clusters' increase of serotonergic and dopaminergic bindings (e.g., Kontis and Theochari, 2012; Kaye et al., 2013; Riva, 2016), particularly in right frontal-temporal regions (Bailer et al., 2007; Galusca et al., 2008).

Despite the marked increase in neurobiological data related to AN, there is still a lack of targeted brain-directed treatment interventions. Most convincing evidence comes from studies applying deep brain stimulation across a variety of brain targets involved in the hypothalamic–mesocorticolimbic pathways. Potential anatomical targets were those involved with reward, cognitive control, motivation, and the learning/memory circuits, such as the ventral tegmental area, the nucleus accumbens and caudate, the subgenual cingulate cortex, the amigdala, the hypppocampus, the insula, and the ventral striatum (for review see McClelland et al., 2013; Lee et al., 2018).

Conversely, the exiting studies that have investigated the efficacy of transcranial non-invasive brain stimulation for reducing AN symptomatology and related behaviors (e.g., Kamolz et al., 2008; McClelland et al., 2013, 2016a,b; Van den Eynde et al., 2013; Khedr et al., 2014), have typically targeted the pre-frontal cortex (PFC), mainly the DLPFC. The DLPFC has a key node of the brain's frontostriatal cognitive circuits, important for inhibitory cognitive control (Miller and Cohen, 2001; Hare et al., 2009), self-control in a dietary context (Wagner et al., 2010; Lowe et al., 2014) and for higher-order reward processing, as part of its involvement in the mesocortical dopaminergic pathway (Diana, 2011), linked to regulation of food intake (Doherty et al., 2016).

Two forms of non-invasive neuromodulatory techniques used in the treatment of AN were transcranial magnetic stimulation (TMS) and transcranial direct current stimulation (tDCS). Both TMS and tDCS have been proven to reduce AN-related behaviors and thoughts (McClelland et al., 2013; Lee et al., 2018). The TMS studies have mostly involved high-frequency (excitatory) TMS to the left PFC. For example, a reduction in levels of feeling full, feeling fat and anxiety has been reported when a single TMS session was applied to the left DLPFC in cases with AN (Van den Eynde et al., 2013; McClelland et al., 2016a,b). Moreover, significant improvement in depressive symptomology and BMI (Kamolz et al., 2008) was described in a case report of 41 sessions of left DLPFC excitatory rTMS, as well as improvement in AN symptomatology and affective symptoms has reported in three of five patients, after 20 sessions of excitatory left DLPFC rTMS. However, such improvement was not always translated into weight gain (McClelland et al., 2016a,b).

Despite recent findings, showing symptoms reduction following TMS excitation of the left DLPFC, one study directly investigated the effects of tDCS on the AN symptomatology (Khedr et al., 2014). Specifically, in this study, it was documented an improvement in AN and in associated depression following 10 daily anodal tDCS (2 mA) sessions of 25 min over the left DLPFC (cathode in extracephalic montage) in five of seven patients immediately post-treatment and in three of seven patients at 1 month follow up.

Conversely, tDCS research has mostly focused on food cravings (Fregni et al., 2008; Goldman et al., 2011; Montenegro et al., 2012; Boggiano et al., 2017) and evidences have been provided that food cravings is reduced when excitatory (anodal) tDCS is applied over the right DLPFC (as opposite to reports on AN where excitatory stimulation is applied over the left DLPFC). For example, Fregni et al. (2008) compared both tDCS protocols, anode right/cathode left and anode left/cathode right, to sham stimulation, and found that food cravings reduced, remained stable or increased in these conditions, respectively. Moreover, both Goldman et al. (2011) and Montenegro et al. (2012) found reduced food cravings and desire to eat in overweight individuals following single session of right anodal/left cathodal tDCS compared to sham. Similarly, Ljubisavljevic et al. (2016) demonstrated that repeated sessions of right anodal/left cathodal tDCS increase the single sessions effect, byreducing the intensity of food craving and the habitual experiences of food craving in individuals with frequent food craving. These studies ground on the evidence that hyperphagia, and more in general craving and substance abuse, is associated with right frontal hypoactivation (Isern, 1987; Fisher et al., 1994; Miller and Cohen, 2001), an opposite pattern of inter-hemispheric imbalance found in AN—i.e., right frontal hyperactivation and dominance (Grunwald et al., 2004; Brooks et al., 2011; Bär et al., 2013; Phillipou et al., 2015).

Bilateral prefrontal cortex tDCS, aimed at rebalancing such inter-hemispheric imbalance by increasing excitability of right hemisphere and decreasing excitability of left hemisphere (the opposite of what is proposed for AN), showed positive findings in different disorders of craving and substance abuse. In particular, left cathodal/right anodal tDCS increased the abstinent rate (Klauss et al., 2014) and reduced the craving in alcoholic patients, with more negative processing of alcohol-related cues after treatment (Wietschorke et al., 2016).

However, in healthy individuals, Vierheilig et al. (2016) aimed at investigating the effects of bilateral tDCS with different electrode montages, on the interaction of attention and emotion processes, found that only left cathodal/right anodal tDCS leads to increase visual attention but neither left cathodal/right anodal or left anodal/right cathodal did influence emotional processing.

Increased knowledge on the role of hemispheric lateralization in ED, together with improvements in the design of neuromodulation protocols, is likely to emerge from studies involving tDCS. tDCS applied on DLPFC should act on the right hyperactivity reported in AN to balancing the activity in both hemispheres.

Considering the paucity of effective treatments for AN, and the increasing incidence rate of AN among children and adolescents, with common long-term physical and psychosocial disability outcomes as well as life risk (Arcelus et al., 2011; Smink et al., 2012; Walsh, 2013), the study was aiming at proving the effectiveness of a non-invasive neuromodulation treatment by tDCS in improving the outcome of traditional treatment in developmental populations with AN.

The safety and tolerability of tDCS in children and adolescents has been proven (Antal et al., 2017) so, in a single-blind-controlled study, we hypothesized that excitatory tDCS over the left DLPFC and inhibitory tDCS over the right DLPFC (anode left/cathode right) may aid in altering/resetting inter-hemispheric balance in adolescents with AN, re-establish their control over eating behaviors. This montage was based on the dominance hypothesis in AN and in accordance with previous studies, where opposite stimulation montage showed an improvement in “over-eating” for ED symptoms (Fregni et al., 2008; Goldman et al., 2011; Montenegro et al., 2012; Boggiano et al., 2017).

## Materials and methods

### Participants

Twenty-three adolescents with diagnosis of AN participated in the study. Eleven participants (10 females; 1 males; mean age = 13.9, *SD* = 1.8 years, range 10.3–15.6; mean IQ = 102.2, *SD* = 4.9, range 95–108; mean BMI = 14.7, *SD* = 2.2, range 11.8–17.5) received an experimental treatment with tDCS additionally to the classical treatment called “as usual” (AU). Twelve participants (12 females; mean age = 15.1, *SD* = 1.5 years, range 13.1–17.8; mean IQ = 100.5, *SD* = 4.6, range 92–106; mean BMI = 15.5, *SD* = 1.6, range 12–17.9) received a Family Based Therapy (FBT) additionally to the AU. All participants received a diagnosis of AN based on the DSM-5 criteria (American Psychiatric Association, 2013). The principal eligibility criterions required were an age between 10 and 17 years, a BMI between 12 and 18 kg/m^2^ and IQ ≥ 85. None had a personal history of neurological disease or a family history of epilepsy and none had comorbidity with other clinically relevant disorders. Both groups did not differ for chronological age [*t*_(21)_ = −1.71, *p* = 0.10], IQ (Raven, 1994) [*t*_(21)_ = 0.85, *p* = 0.41] and BMI [*t*_(21)_ = −1.01, *p* = 0.32].

All participants received Atypical antipsychotics (AA) as pharmacological treatment, in particular Aripiprazole. Some of them also received SSRIs (5 in the tDCS group and 9 in the FBT group) or Benzodiazepines (Benz: 2 in the tDCS group and 1 in the FBT group). The two groups did not differ in the frequency of each pharmacological treatment [$\chi_{\left(2\right)}^{2}$ = 2.10, *p* = 0.35].

Written informed consent was obtained from all participants and their parents after the procedures had been fully explained.

### Materials

#### Outcome measures

All participants were evaluated through clinical tests assessing psychopathological conditions. The AN symptomatology assessment included: Eating Disorder Inventory (Giannini et al., 2008; EDI-3, Garner, 2004), Eating Attitudes Test (EAT-26, Garner et al., 1982), and Body Uneasiness Test (BUT, Cuzzolaro et al., 2006). The EDI-3 is a self-report questionnaire, including 91 items divided into 12 subscales rated on a 0–4 point scoring system. It gives a measure of the basic characteristics of the eating disorder. The EAT-26 is a forced choice, self-report questionnaire, including 40 items, measuring anorexia nervosa symptoms. The BUT is a 71-item self-report questionnaire which measures body image concerns.

Moreover, anxiety and depressive symptoms were evaluated through the Multidimensional Anxiety Scale for Children (MASC, March et al., 1997) and the Children's Depression Inventory (CDI, Kovacs, 1982, 1992). The MASC is a quantitative self-report scale on anxiety symptoms, age range: 8–19. The CDI is a self-report questionnaire with 27 items, which evaluates the mood symptoms in the last 2 weeks, age range: 8–17. All clinical questionnaires were completed by the participants themselves. In addition, anthropometric measures, as BMI, were measured.

#### Safety and tolerability

Side-effects of tDCS were assessed by a standard questionnaire (Brunoni et al., 2011) which was completed by participants after each stimulation session. The questionnaire lists adverse effects, such as headache, neck pain, scalp pain, tingling, itching, burning sensation, skin redness, sleepiness, trouble concentrating, and acute mood change. Participants quantify the intensity of the symptoms or side-effects related to tDCS (1, absent; 2, mild; 3, moderate; 4, severe).

### Procedures

All participants underwent the treatment AU, including nutritional, pharmacological, and psychoeducational treatment. The experimental group received an add-on treatment with 18 sessions of tDCS (TDCS+AU) for 6 weeks, while the control group received the FBT (FBT+AU).

All participants have not received the AU, the FBT, or the tDCS treatment previously. Outcome measures were assessed before (T0) and immediately after the end of treatments (T1), i.e., 6 weeks later. Nine of the eleven participants of the tDCS+AU group were followed-up 1 month after the end of treatment (T2).

#### Treatments

##### tDCS

In the tDCS condition all the participants received 1mA tDCS. Direct current was applied for 20 min, 3 times a week for 6 weeks (18 sessions). Anodal electrode was positioned over the left DLPFC and cathodal electrode over the right DLPFC according to the 10–20 EEG system and the sites corresponding respectively at F3 and F4 area. The selected montage (anodal/left—cathodal/right) was applied to re-balance the right frontal hyperactivity reported in literature in individuals with AN (Grunwald et al., 2004; Brooks et al., 2011; Bär et al., 2013; Phillipou et al., 2015). Direct current was generated by a BrainStim stimulator by E.M.S. s.r.l. (Bologna, Italy). It was delivered on the scalp via a pair of identical, rectangular, electrodes (5 × 5 cm) covered with conductive rubber and saline soaked synthetic sponges. During the tDCS sessions, all participant have been sat in a comfortable chair.

##### AU

Each participant undergone the treatment AU treatment during the study. In this study, after a first assessment whit a psychiatric interview, an interview for family diagnosis and nutritional monitoring, participants and parents received a psychological support. Furthermore, meetings for the nutritional and psychiatric monitoring for patients were given (once every 2 weeks). Psychological support for patients were given by individual sessions (once every 2 weeks, 60 min duration) and group sessions (once every week, 60 min duration). Their parents received the psychoeducation in group sessions (once every 2 weeks, 60 min duration).

##### FBT

In this group, participants and parents received family psychotherapy provided by professionals (psychotherapists and psychiatrists), additionally to the AU treatment. Weekly sessions of participants' group therapy and parents' group therapy were delivered while, family meetings occurred every 15 days.

### Data analysis

To evaluate the effect of treatment, repeated measures ANOVAs were performed on each psychopathological measure (EDI-3; EAT-26; BUT; CDI; MASC) and on BMI values, with Group (tDCS+AU vs. FBT+AU) as between subjects factor and Time (T0, T1) as within subjects factor.

The demographic variables and the baseline measures were compared through the Student's *t-*Test for independent samples. Chi-squared test was used to value the non-parametric variables. Post hoc analyses were performed by the Tukey's test. In the tDCS group the follow-up data were analyzed through the Wilcoxon signed-rank test. Partial eta squares (η_*p*_^2^) have been reported as effect size measures. A *p*-value less to 0.05 was considered as statistically significant. Sphericity was verified by Mauchly's sphericity test. The Pearson correlation was used to test the association between the outcome of improvement [(T1-T0/T0)^*^100] on BMI and the other psychopathological measures.

### Ethic approval

This study was performed in accordance with the World Medical Association's Declaration of Helsinki and The Research Ethical Committee of the Bambino Gesù Children's Hospital approved this study under process number 763-OPBG-2014.

### Results

#### Baselines measures

tDCS+AU and FBT+AU groups did not differ for BMI as well as for baseline psychopathological measures: EDI-3 [*t*_(21)_ = −1.14, *p* = 0.27], EAT-26 [*t*_(21)_ = −0.70, *p* = 0.49], BUT [*t*_(21)_ = −1.33, *p* = 0.20], MASC [*t*_(21)_ = 1.11, *p* = 0.28], CDI [*t*_(21)_ = 0.46, *p* = 0.65]. Baseline psychopathological measures did not significantly differ between groups even on subscales of EDI-3 and MASC. As concerns EDI-3 subscales: Drive for Thinness [*t*_(21)_ = −1.23, *p* = 0.23], Bulimia [*t*_(21)_ = −0.66, *p* = 0.52], Body Dissatisfaction [*t*_(21)_ = −0.76, *p* = 0.46], Eating Disorder Risk [*t*_(21)_ = −1.0, *p* = 0.33], Low Self-Esteem [*t*_(21)_ = −0.61, *p* = 0.55], Personal Alienation [*t*_(21)_ = −0.51, *p* = 0.62], Interpersonal Insecurity [*t*_(21)_ = −0.70, *p* = 0.50], Interpersonal Alienation [*t*_(21)_ = −1.11, *p* = 0.28], Interoceptive Deficits, [*t*_(21)_ = −0.98, *p* = 0.34], Emotional Dysregulation [*t*_(21)_ = −0.77, *p* = 0.45], Perfectionism [*t*_(21)_ = −1.94, *p* = 0.07], Asceticism [*t*_(21)_ = −0.17, *p* = 0.86], Maturity Fears [*t*_(21)_ = −0.44, *p* = 0.67], Ineffectiveness [*t*_(21)_ = −0.73, *p* = 0.47], Interpersonal Problems [*t*_(21)_ = −1.05, *p* = 0.31], Affective Problems [*t*_(21)_ = −1.24, *p* = 0.23], Overcontrol [*t*_(21)_ = −1.08, *p* = 0.29], Global Psychological Maladjustment [*t*_(21)_ = −1.55, *p* = 0.14]. As concerns MASC subscales: Physical Symptoms [*t*_(21)_ = 0.67, *p* = 0.51], Harm Avoidance [*t*_(21)_ = 0.74, *p* = 0.47], Social Anxiety [*t*_(21)_ = 0.58, *p* = 0.57], Separation/Panic [*t*_(21)_ = 0.03, *p* = 0.97], Adi [*t*_(21)_ = 0.80, *p* = 0.43].

Means and Standard Deviations per each measure are shown on Table 1.

**Table 1:** Mean (standard deviation) per measure of the main effect of time and of the interaction time per group.

	**Time**					**Time X Group**						
**Measures**						**tDCS** + **AU**		**FBT** + **AU**				
	**T0**	**T1**	***F^a^***	***p***	**η*_*p*_*^2^**	**T0**	**T1**	**T0**	**T1**	***F^a^***	***p***	**η*_*p*_*^2^**
	**M (*SD*)**	**M (*SD*)**				**M (*SD*)**	**M (*SD*)**	**M (*SD*)**	**M (*SD*)**			
BMI	15.1 (1.9)	16.3 (1.8)	35.78	<**0.001**	0.42	14.7 (2.2)^b^	16.6 (2.3)^b*^	15.5 (1.6)	16.1 (1.3)	9.75	<**0.01**	0.32
EDI-3 TOT	66.6 (17.1)	52.6 (21.6)	7.92	**0.01**	0.28	62.4 (21.0)	48.9 (24.9)	70.7 (13.3)	56.2 (18.3)	0.05	0.82	< 0.01
Drive for thinness	75.0 (23.1)	53.7 (35.3)	10.01	<**0.01**	0.34	68.6 (33.6)	44.7 (40.3)	81.4 (12.5)	62.7 (30.3)	0.14	0.71	< 0.01
Bulimia	45.1 (33.1)	34.1 (31.9)	1.56	0.23	0.07	40.4 (32.6)	31.9 (31.4)	49.7 (33.5)	36.4 (32.4)	0.08	0.78	< 0.01
Body dissatisfaction	75.5 (19.8)	61.5 (29.8)	7.77	**0.01**	0.28	72.3 (21.5)	55.8 (30.8)	78.7 (18.1)	67.2 (28.9)	0.25	0.62	0.01
Eating disorder risk	73.4 (19.9)	55.6 (29.6)	10.73	<**0.01**	0.35	69.1 (26.0)	49.1 (32.5)	77.7 (13.8)	62.1 (26.8)	0.16	0.69	< 0.01
Low self-esteem	69.2 (24.6)	49.8 (31.7)	9.18	<**0.01**	0.31	66.0 (29.5)	48.3 (34.5)	72.4 (19.7)	51.4 (29.0)	0.01	0.80	< 0.01
Personal alienation	68.6 (28.8)	47.9 (28.7)	15.19	<**0.001**	0.43	65.5 (30.6)	41.3 (34.5)	71.7 (27.0)	54.6 (23.0)	0.44	0.52	0.02
Interpersonal insecurity	65.1 (24.0)	53.0 (29.0)	5.24	**0.04**	0.21	61.5 (23.2)	44.6 (27.3)	68.7 (24.8)	61.4 (30.8)	0.84	0.37	0.04
Interpersonal alienation	62.8 (28.4)	44.9 (32.4)	6.72	**0.02**	0.25	56.1 (31.0)	41.2 (33.3)	69.5 (25.8)	48.7 (31.6)	0.19	0.67	< 0.01
Interoceptive deficits	69.7 (25.3)	58.6 (30.9)	2.50	0.13	0.11	64.4 (23.3)	54.5 (32.1)	75.1 (27.3)	62.7 (29.7)	0.03	0.86	< 0.01
Emotional dysregulation	66.7 (34.7)	51.6 (32.0)	1.39	0.25	0.04	65.4 (32.2)	46.9 (32.3)	68.0 (37.2)	56.4 (31.8)	0.01	0.91	< 0.001
Perfectionism	56.8 (27.7)	52.9 (28.7)	0.35	0.56	0.02	45.3 (34.3)	51.6 (22.2)	68.4 (21.1)	54.3 (35.3)	2.44	0.13	0.11
Asceticism	72.5 (27.1)	55.1 (29.4)	5.17	**0.03**	0.20	71.5 (30.9)	60.2 (34.6)	73.5 (23.3)	50.1 (24.2)	0.63	0.44	0.03
Maturity fears	69.1 (29.8)	66.5 (27.5)	0.15	0.70	< 0.01	66.3 (29.1)	65.3 (19.5)	71.9 (30.5)	67.7 (35.5)	0.06	0.82	< 0.01
Ineffectiveness	70.2 (25.7)	50.0 (29.8)	12.91	<**0.01**	0.39	66.2 (30.1)	45.1 (34.5)	74.2 (21.3)	54.9 (25.2)	0.02	0.88	< 0.01
Interpersonal problems	66.0 (25.6)	50.0 (30.3)	8.81	<**0.01**	0.31	60.3 (26.7)	42.9 (31.9)	71.7 (24.6)	57.2 (28.7)	0.07	0.80	< 0.01
Affective problems	67.3 (30.3)	57.0 (30.9)	1.87	1.87	0.09	59.3 (29.1)	52.6 (32.4)	75.4 (31.5)	61.4 (29.4)	0.23	0.64	0.02
Overcontrol	67.7 (25.9)	58.0 (25.6)	1.99	0.17	0.09	61.8 (29.5)	59.9 (27.7)	73.7 (22.3)	56.2 (23.6)	1.29	0.27	0.06
Global psychological maladj	74.4 (20.7)	58.0 (26.3)	7.91	**0.01**	0.28	67.5 (26.2)	52.6 (30.8)	81.3 (15.2)	63.4 (21.9)	0.07	0.80	< 0.01
EAT-26	39.5 (19.6)	23.4 (19.1)	18.04	<**0.001**	0.46	36.6 (19.2)	24.2 (22.7)	42.4 (20.1)	22.7 (15.5)	0.93	0.35	0.04
BUT	2.3 (1.3)	2.0 (1.4)	2.36	0.14	0.10	2.0 (1.1)	1.8 (1.4)	2.7 (1.3)	2.2 (1.4)	0.20	0.66	< 0.01
MASC TOT	54.5 (12.1)	49.3 (10.8)	7.58	**0.01**	0.26	57.3 (13.0)	52 (11.3)	51.7 (11.2)	46.7 (10.3)	< 0.01	0.94	< 0.001
Physical symptoms	56.4 (8.7)	49.6 (9.1)	15.61	<**0.001**	0.43	57.7 (8.6)	50.3 (9.5)	55.2 (8.9)	48.9 (8.7)	0.10	0.75	< 0.01
Harm avoidance	44.4 (10.9)	41.6 (10.8)	1.67	0.21	0.07	46.1 (10.2)	44.9 (9.7)	42.7 (11.7)	38.3 (11.9)	0.55	0.46	0.03
Social anxiety	57.1 (12.2)	54.2 (10.5)	1.86	0.18	0.08	58.6 (13.5)	56.7 (13.2)	55.7 (11.0)	51.7 (7.9)	0.22	0.64	0.01
Separation/Panic	50.5 (9.2)	50.0 (9.5)	0.10	0.75	< 0.01	50.6 (6.1)	52.0 (9.0)	50.5 (12.4)	48.1 (10.0)	1.34	0.26	0.06
Adi	51.5 (12.3)	45.7 (12.5)	5.57	**0.03**	0.21	53.6 (12.4)	50.4 (12.0)	49.4 (12.2)	41.0 (13.0)	1.14	0.30	0.05
CDI	22.0 (11.7)	12.3 (7.95)	24.30	<**0.001**	0.54	23.2 (15.5)	11.4 (9.5)	20.8 (7.9)	13.2 (6.4)	1.16	0.29	0.05

a df = 1, 21.

b *Means in the same row with same subscripts are significantly different*.

* p < 0.001.

Significant values are highlighted in bold.

*T0, baseline or before treatment; T1, after treatment; Groups: tDCS + AU = tDCS + As Usual; FBT + AU = Family Based Therapy + As Usual; Maladj, maladjustment*.

### Post-treatment measures

#### BMI

**Figure 1:**
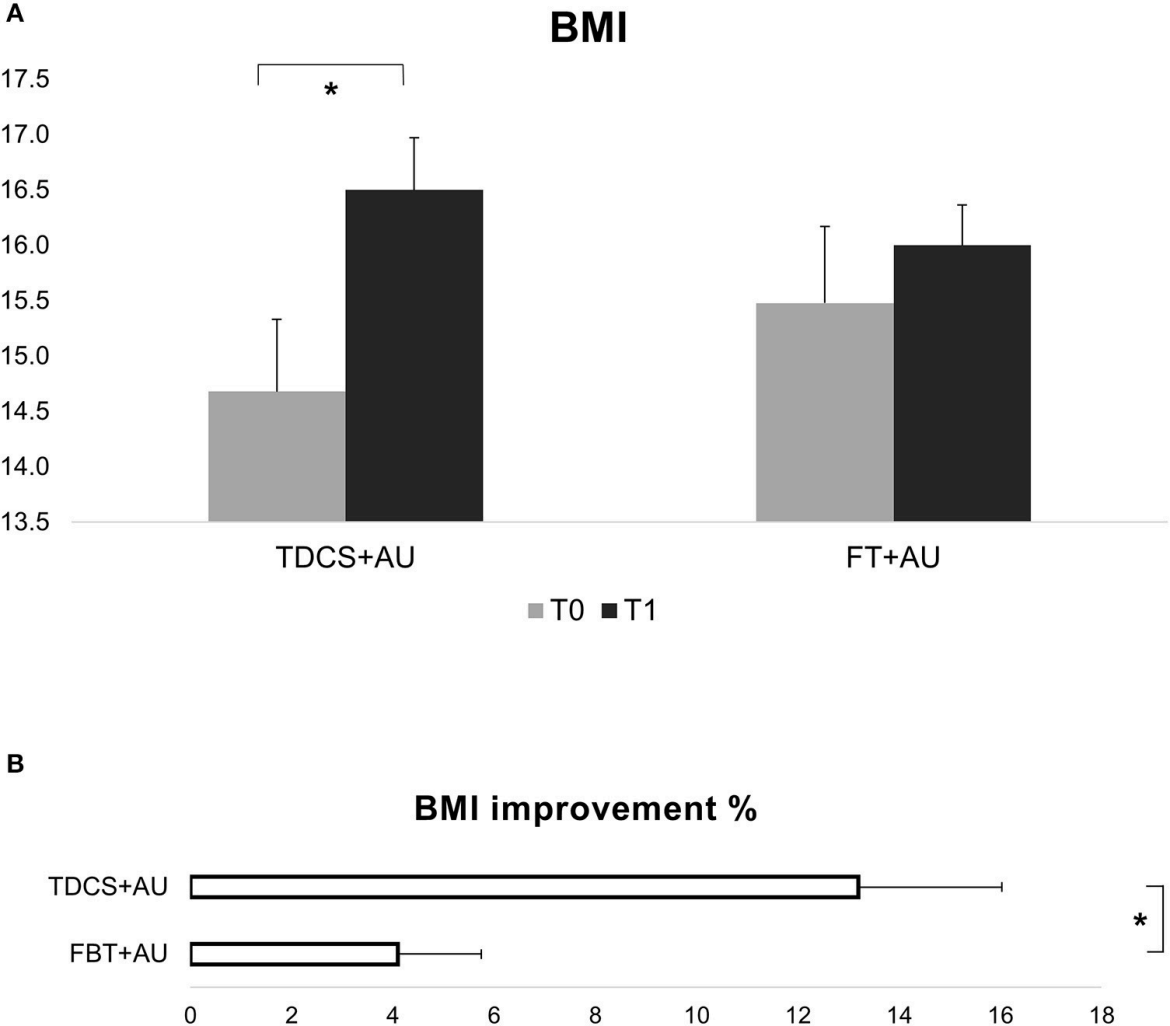
**(A)** Shows BMI values expressed in mean at baseline (T0) and after the end of treatment (T1). In the tDCS+AU group BMI values increased significantly more than in the FBT+AU group. **(B)** Shows the percentage of increase [(T1-T0/T0)*100]: in the tDCS+AU group it amounted to 13.3% (±9.4) while in the FBT+AU group it amounted to 4.2% (±5.7). *p < 0.05.

Results showed a significant interaction between Group and Time [*F*_(1, 21)_ = 9.75; *p* < 0.01, η_*p*_^2^ = 0.32]. BMI improved significantly in the tDCS+AU (*p* < 0.001), while in the FBT+AU no difference emerged in BMI after treatment compared to baseline (*p* = 0.2). Figure 1A, shows the mean BMI values in both groups: at baseline (pre-treatment) and after 18th session (post-treatment). The mean percentage of BMI improvement [(T1-T0/T0)^*^100] in the tDCS+AU group amounted to 13.3% (±9.4) while in the FBT+AU group it amounted to 4.2% (±5.7). See Figure 1B.

**Figure 2:**
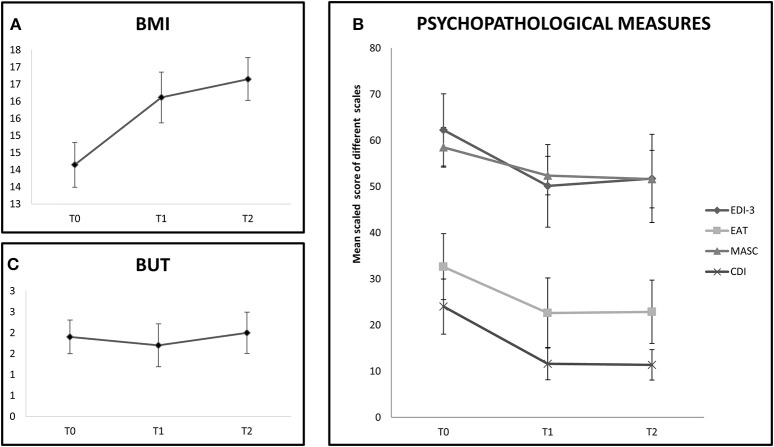
The chart shows follow-up evalution of outcome measures in the tDCS+AU group (only 9 participants). **(A)** Shows BMI values expressed in mean at baseline (T0), at the end of treatment (T1) and at 1 month follow-up (T2). BMI values increased even at 1-month follow-up. **(B)** Shows mean scaled score of EDI-III, EAT-26, MASC, and CDI at baseline (T0), at the end of treatment (T1) and at 1 month follow-up (T2). Improvement was stable until 1-month follow-up in the EAT-26, MASC, and CDI scales, but not on EDI-III. **(C)** Shows mean score of BUT at baseline (T0), at the end of treatment (T1) and at 1 month follow-up (T2). No improvement was shown at any evaluation time.

In the tDCS group the BMI improvement persisted also 1 month later (T0 vs. T2: *Z* = 2.66, *p* < 0.01). See also Figure 2A.

A general Group effect did not emerged [*F*_(1, 21)_ = 0.05; *p* = 0.83, η_*p*_^2^ = 0.01] while emerged a main effect of Time [*F*_(1, 21)_ = 35.78; *p* < 0.001, η_*p*_^2^ = 0.42]. See Table 1.

#### Psychopathological assessment

In the psychopathological scales there were no significant interaction between Group and Time (all comparisons *p* > 0.1). No main Group effect emerged in each measure (all comparisons *p* > 0.1). A Time effect was significant independently of the group, in most of the comparison: both groups improved in the clinical scales EDI-3, MASC, and CDI with the exception of BUT. Moreover, both groups improved in most subscales of EDI-3 and MASC. In particular, as concerns EDI-3 subscales, independently of the group, a mean score reduction was observed after treatment on Drive for Thinness, Body Dissatisfaction, Eating Disorder Risk, Low Self-Esteem, Personal Alienation, Interpersonal Insecurity, Interpersonal Alienation, Asceticism, Ineffectiveness, Interpersonal Problems and Global Psychological Maladjustment (all *p* < 0.05). However no improvement emerged on the subscales Bulimia, Interoceptive Deficits, Emotional Dysregulation, Perfectionism, Maturity Fears, Affective Problems, and Overcontrol (all *p* > 0.10). As concerns MASC subscales, independently of the group, a mean score reduction was observed after treatment on Physical Symptoms and Adi (all *p* < 0.05) while no improvement emerged on Harm Avoidance, Social Anxiety, and Separation/Panic (all *p* > 0.10).

Table 1 shows the total score on these scales and their subscales in both groups at baseline (pre-treatment) and after 18 session (post-treatment), as well as the statistics for the main effect of Time and for the interaction effect of Time per Group.

In the tDCS+AU group, which was followed-up for 1 month, the effect on most of the psychological measures persisted 1 month after the end of treatment (EAT-26, T0 vs. T2: *Z* = 2.37, *p* = 0.02; MASC, T0 vs. T2: *Z* = 1.96, *p* = 0.05; CDI, T0 vs. T2: *Z* = 2.19, *p* = 0.04), but not on EDI-3 (T0 vs. T2: *Z* = 1.33, *p* = 0.18). Again no improvement was observed on BUT even after 1 month (T0 vs. T2: *Z* = 0.56, *p* = 0.57). See Figures 2B,C.

#### Correlation between outcomes

In the tDCS+AU group we found a medium negative correlation between the improvement on BMI and the score changes on EDI-3 (*r* = −0.65, *p* = 0.04), i.e., the higher the BMI increased (amelioration), the lower the EDI-3 was after treatment (amelioration). However, we failed to found such correlation in the FBT+AU group (*p* = 0.29).

By considering the EDI-3 subscales, in the tDCS+AU group we found a medium negative correlation between the improvement on BMI and the score changes on Bulimia (*r* = −0.68, *p* = 0.03) and on Global Psychological Maladjustment (*r* = −0.67, *p* = 0.03). Moreover we found a strong negative correlation between the improvement on BMI and the score changes on Interpersonal Problems (*r* = −0.71, *p* = 0.02). However, we did not found any correlation between the BMI improvement and other EDI-3 subscales score changes (all *p* > 0.1). Conversely, by considering the EDI-3 subscales, in the FBT+AU group we found a medium positive correlation between the improvement on BMI and the score changes on Low Self-Esteem (*r* = 0.60, *p* = 0.04), i.e., the higher the BMI increased (amelioration), the higher the Low Self-Esteem score was after treatment (worsening).

Finally, we did not found any correlation between the improvement on BMI and the other psychopathologiocal scales or subscales, neither in the tDCS+AU group nor in the FBT+AU group (all *p* > 0.1).

## Safety and tolerability

Concerning safety and tolerability, no participant asked to stop the study or reported significant discomfort at the electrode sites. Participant tolerated tDCS well. The most frequent adverse effects were itching sensation, burning sensation (reported by 9 participant), especially in the first seconds of stimulation, which diminished rapidly with water's addition under the sponge and local redness (report by 8 participant). Others effects were headache (reported by 5 participants), tingling (reported by 5 participants) and, mostly in the mild intensity (see Table 2).

**Table 2:** Percentage of adverse effects reported by participants.

**Adverse effect**	**Mild (%)**	**Moderate (%)**	**Severe (%)**
Headache	18.1	27.2	0.0
Neck pain	0.0	0.0	0.0
Scalp pain	0.0	0.0	0.0
Tingling	45.4	0.0	0.0
Itching	54.5	27.2	0.0
Burning sensation	27.7	27.7	9.0
Local redness	36.3	45.4	0.0
Sleepiness	0.0	0.0	0.0
Trouble concentrating	0.0	0.0	0.0
Acute mood changes	0.0	0.0	0.0
Irritability	0.0	0.0	0.0

Neck pain, sleepiness, and trouble concentrating were not reported. No psychological symptoms, such as acute mood changes and irritability, nor other discomforts or adverse effects were reported.

## Discussion

The present study evaluated the efficacy of a tDCS treatment in a young population with AN.

Two groups were compared: a group received tDCS treatment in addition to “as usual” treatment; another group received an active control treatment, i.e., a family based psychotherapy, in addition to “as usual” treatment.

In the experimental group, tDCS was applied to the DLPFC and, more specifically, anodal electrode was positioned over the left and cathodal electrode over the right area. According to literature, this montage, with concurrent left excitatory and right inhibitory stimulation, was applied to re-balance the right frontal hyperactivity reported in AN (Grunwald et al., 2004; Brooks et al., 2011; Bär et al., 2013; Phillipou et al., 2015). Furthermore, all participants received a nutritional and psychiatric monitoring as well as psychological support.

The main finding was that after 6 weeks of treatment, the tDCS+AU group, but not the control group, showed significant increase of BMI values. Indeed, the mean percentage of BMI increment in the group receiving tDCS amounted to 13.3% compared to 4.2% in the FBT+AU group. Moreover, independently of the treatment, all participants improved in several psychopathological measures, included AN psychopathology and mood and anxiety symptoms, with the exception of the measure assessing body uneasiness (BUT). The psychopathological improvement profile seems to overlap in the two groups, indeed there are no differences in any subscales of each psychopathological measure. In the tDCS+AU group, the BMI increment as well as the improvement in some psychopathological measures persisted even at 1-month follow-up.

Our results confirmed previous findings on AN reporting positive effects in mood and AN symptoms after non-invasive brain stimulation, as described on case reports with tDCS (Khedr et al., 2014), and on group studies with TMS (Kamolz et al., 2008; McClelland et al., 2013, 2016a,b; Van den Eynde et al., 2013). However, present study firstly demonstrated a specific effect of a tDCS treatment on stable weight gain and a superiority compared to an active control treatment for adolescents with AN.

It is important to note that the two groups received similar nutritional, psychoeducational and pharmacological concurrent treatment (the AU treatment), which might have a main role in eating behavior and weight gain, while they differed for the specific add-on treatment (i.e., the experimental tDCS or the treatment of choice FBT). This means that the results obtained can be explained by the add-on treatment or by the interaction between each add-on treatment and the AU treatment.

Literature on clinical trials for AN, strengthens the importance of obtaining BMI improvement, together with the psychopathological improvement, as the optimal outcome for AN remediation (Halmi, 1982; Guarda, 2008). Although weight gain has been consistently found using invasive neuromodulation approaches to AN, such as DBS, evidence from non-invasive brain stimulation indicated an impact on self-reported symptoms but not always translated to weight gain (Lee et al., 2018). Our tDCS study, instead, gives first evidence of the positive effect of a 6 weeks tDCS treatment in improving both the psychopathological symptoms and the MBI in adolescents with AN, compared to a control active treatment, widely considered treatment of choice.

Moreover, our study shows a beneficial association between the improvement in BMI and the amelioration of psychopathological symptomats with the tDCS treatment but not with the control treatment. Specifically, it has been found that, within the experimental group, the increase in BMI was associated to a reduction of EDI-3 total scores (risk of eating disorder) and of Bulimia, Interpersonal Problems and Global Psychological Maladjustment subscales. On the other hand, in the control group, a relation was found between the increase in BMI and the worsening to the Low Self-Esteem subscale.

These results suggests that tDCS may have an action in the improvement of cognitive symptoms linked to incorrect food behavior, as already highlighted by other researchers (McClelland et al., 2013; Lee et al., 2018). In particular, the reduction of the Bulimia subscale score, with the increase of BMI, is particularly relevant because it indicates an improvement in cognitive control, i.e., the reduction of compensatory behaviors in order to prevent weight gain, which is often observed in the weight recovery phases in patients with AN (Bulik et al., 1997; Fairburn and Harrison, 2003). Moreover, the positive effect of tDCS on BMI seems to be accompanied by a more general positive effect on other behaviors, such as interpersonal relationships and psychological adaptation, resulting in an improvement of the risk of eating disorder (reduction of EDI-3 total score).

On the other hand, it is often reported in people with AN that an improvement on weight, but not at the same time on cognitive and behaviors symptoms, is negatively experienced and affects their self-esteem, characteristic trait of the AN disease (Striegel-Moore et al., 2004). This seems the case of the control group, who showed a worsening of self-esteem score with BMI improvement.

All together these results seem to suggest a superior combination of psychological changes associated with weight gain in the experimental group, than in the control group. The combination of weight gain and psychological change is of crucial importance for eating disorders recovery (Bardone-Cone et al., 2010).

The reason why in our study there was a positive and specific effect on BMI, unlike what has been reported so far by non-invasive brain stimulation studies, may depend on the type of stimulation performed. Most of the non-invasive brain stimulation studies used TMS to facilitate left PFC. In comparison with TMS, the mechanism by which tDCS works enables multiple stimulation designs. Switching the position of the electrodes enables swapping of excitation/inhibition between the right and left hemispheres. This may act to balancing the activity in both hemispheres, thus being likely more efficient in resetting the inter-hemispheric balance alterated in AN.

The lone tDCS study (Khedr et al., 2014) was an open label, single-arm study, showing improvement in psychopathology scales, with important variation between seven patients, after 10 sessions of left Anodal tDCS on DLPFC. In particular, five patients improved in all scales, but just three maintained this scores 1 month later. This work included the total scores of the EAT, EDI, and the Beck Depression Inventory. However, the study did not report BMI measures, showing only positive correlations in the percent of improvement between eating disorder scales and depression scale.

Compared to our study, difference in some elements might be relevant. In the Khedr et al.'s study, tDCS was applied for 25 min (2 mA) with an extracephalic cathodal electrode, in a monopolar montage to activate the left DLPC. The study ground on previous TMS literature, using a facilitatory stimulation to the left DLPC to improve AN. Conversely, we used a bilateral montage of electrodes on DLPFC, anodal/left and cathodal/right, with the aim to induce a simultaneously increasing in the left DLPFC activation and reducing in the right DLPFC activation (Nasseri et al., 2015).

We hypothesize that, the relevant BMI increase in the tDCS group may be due to the concurrent delivering of the cathodal stimulation to the right DLPFC, rather than just to the anodal stimulation to the left PFC, for a possible direct/indirect action into some pathophysiological mechanisms of AN, involving the mesocortical dopaminergic pathways and the promotion of food intake.

Cortical tDCS stimulation has been shown to modulate dopamine (DA) release in striatum (Tanaka et al., 2013). In particular, following the application of cathodal, but not anodal tDCS for 10 min, extracellular DA levels, measured with *in vivo* microdialysis, increased for more than 400 min in the in rat striatum. This result suggested that tDCS has a direct and/or indirect effect on the dopaminergic system in the subcortical area. However, there is concern if a low intensity current can go that deep in human brain. Very recently, the first evidence in humans has been published (Fonteneau et al., 2018) that bifrontal tDCS induces neurotransmitter release in subcortical areas. Specifically, left anodal/right cathodal tDCS (our study montage) induced a significant increase in extracellular dopamine in a part of the striatum involved in the reward–motivation network.

Mesolimbic dopaminergic projections into striatum are hypothesized to play a key role in governing eating behavior, by modulating appetitive motivational processes. A perturbations in the dopaminergic reward pathways has been hypothesized to play a role in the AN pathogenesis (Casper, 2006; Alcaro et al., 2007). Those alteration included reduced Cerebro Spinal Fluid levels of DA metabolites (Kontis and Theochari, 2012), functional DA D2 receptor gene polymorphisms (Bergen et al., 2005), and increased D2/D3 receptor binding in the striatum (Kaye et al., 2013; Riva, 2016), indicating an increased D2/D3 densities and a decreased extracellular DA in this region. Moreover, the evidence of a DA imbalance in the ventral striatum in patients with AN, is considered to contribute to anhedonia of feeding behavior, ascetic and anhedonic temperament (Frank et al., 2017), as well as dysphoric mood and anxiety (Kaye et al., 2009). Although these findings on the role of DA in AN is still a matter of debate (Gillman and Lichtigfeld, 1986; Kontis and Theochari, 2012).

A possible interpretation of such contrasting results is in the dissociation in the pattern of tonic and phasic firing of dopaminergic signaling (Kontis and Theochari, 2012). Preclinical data show that average extracellular DA in the nucleus accumbens is low in AN and largely arises from phasic DA transients (Owesson-White et al., 2012). Response to behaviorally relevant stimuli triggers the phasic component of DA release onto postsynaptic targets, while the tonic DA levels are proposed to regulate the amplitude of the phasic DA response.

It is believed that glutamate mainly from prefrontal projections (which is usually increased by tDCS) promotes tonic release of DA in the dorsal striatum and in the nucleus accumbens (Grace, 1991; Södersten et al., 2016). Grace (1991) proposed a model for which a pathological alteration in the PFC may cause a reduction in the tonic levels of DA, resulting in excess response of the phasic dopaminergic system and to an increase in the responsiveness of the dopaminergic system.

An increase in the responsiveness of the dopaminergic system has been indeed reported in AN (for a review see Södersten et al., 2016). One might argue that if the dopaminergic system is hypersensitive in AN, it should promote food seeking instead of avoidance. Research that investigated those phenomena has suggested that dopaminregic hypersensitivity in AN is not meant to imply that the so called “reward stimuli” (such as sugar solution) are necessarily a reward in the sense of positive reinforcer or pleasant experience for individuals with anorexia nervosa (Frank et al., 2017). Rather brain dopaminergic circuits in AN could be hypersensitive to salient stimuli in general, including both rewarding or punishing stimuli (Frank et al., 2017). Such failure to appropriately bind, modulate or discriminate responses to stimuli has been taken into account to explain the role of starvation as a reward stimulus and the negative emotional activity in front of food stimuli (Berridge and Robinson, 1998; Frank et al., 2017).

It is therefore possible to hypothesize, albeit only speculatively, that our left anodal/right cathodal tDCS treatment, aimed at rebalancing the hyperactivity of right DLPC, may have aid in restoring the cortical glutamatergic system regulating DA tonic relies in striatum, thus in turn acting in rebalancing the dopaminergic alterations seen in the reward brain network in AN, crucial for regulate the food intake behavior.

A possible confound in our study is the interaction of tDCS with the concurrent pharmacological treatment (Nitsche and Paulus, 2000; Nitsche et al., 2003, 2006, 2012; Monte-Silva et al., 2009; Ridding and Ziemann, 2010; Fresnoza et al., 2014). In particular, there is evidence that SSRIs may themselves increase the response to tDCS (Normann et al., 2007).

Conversely, medications that interfere with dopaminergic signaling, such as antipsychotics, are likely to have a negative impact on tDCS plasticity (Monte-Silva et al., 2009). However, such impact is related to the pharmacological profile of the antipsychotic medication. In particular, the availability of D2 receptors is vital for potentially adaptive neuroplastic effects of tDCS (Nitsche et al., 2009). Aripiprazole, the medication received by all participants, differently from other antipsychotic agents, it is not a pure antagonist at the D2 receptor but a partial agonist (Ziadi Trives et al., 2013). Hence, the drug molecule allows the physiological activity of these receptors, which in turn allows for the beneficial neuromodulatory effects of tDCS. Indeed, tDCS in conjunction with Aripiprazole (but not with other antipsychotics) have proven to improve schizophrenic symptoms (Agarwal et al., 2015).

These considerations lead us to suppose that, the interaction of tDCS with the concurrent pharmacological treatment may have increased, rather than reduced, the plasticity induced effect of our stimulation protocol. Moreover, Aripiprazole may have specifically improved and amplifying the hypothesized tDCS action in regulating the DA tonic component in striatum, given that the drug acts suppressing the phasic component, while relatively preserving the release of the tonic component of DA (Hamamura and Harada, 2007). A positive effect of Aripiprazole on the dopaminergic reward circuit alterations has been recently reported in patients with AN, resulting in an increasing in BMI values (Frank et al., 2017).

So that, it is plausible that the positive effects found on both BMI and psychopathological symptoms, may arise by a synergistic action between a cortical stimulation and a medical stimulation in regulating the imbalance between the tonic and phasic component of DA in AN.

In summary, it is possible to speculate that tDCS treatment may have a direct/indirect action to one of the etiopathogenetic mechanism of AN and may represent a more targeted perspective treatment for AN, especially in adolescence age. Indeed, tDCS has the potential to timely target brain abnormalities through brain plasticity mechanisms, essential in development. Actually, although “malleable” during the early stages, once established, AN are remarkably persistent (Walsh, 2013), therefore it is essential to timely treat the disorder.

Our results are promising, since participants in the tDCS group have increased by almost tri-times their BMI values compared to the participants receiving the AU treatment (both pharmacological, nutritional and psychoeducational) and the psychological treatment of choice. Moreover, our participants did not experience any notable symptoms or side effects (for Brunoni's standard questionnaire, Brunoni et al., 2011) after any stimulation session, thus confirming a high tolerability and feasibility of a tDCS treatment in children and adolescents with AN.

However, an important limitation to these data is that this study was an open label study with an active control group but not a sham control group, therefore any effect cannot be distinguished from a possible tDCS placebo effect. However, given the difficult in enrolling adolescents with AN, which are usually reluctant to receive any kind of treatment, this study serve as a pilot, with preliminary results, to lay the groundwork for a clinical trial.

Undoubtedly, new studies are required with double blind randomized clinical trials, larger sample, and longer follow-ups to confirm our results. Nevertheless, our promising results deserve to be followed by future studies with more advanced analysis, with insight from functional neuroimaging and animal studies, to deeper understand the role of left anodal/right cathodal tDCS to the DLPFC in potentially contrasting some etiopathogenetic mechanisms of AN. If the tDCS treatment will be confirmed to be effective, it may lead to important changes in the treatment of AN which could be translated into novel and effective rehabilitation strategies, especially in developmental age.

## Author contributions

All authors provided substantial contributions to the work. FC, DM, and SV conceived and designed the study. FC and AnM conducted experimental treatment. VZ, MC, and AlM performed the measurements, contributed to sample preparation and to follow up. FC, DM, and AnM drafted the manuscript. SV, AlM, DM, and VZ revised the manuscript. After revisions and editing by all authors, the article was submitted.

### Conflict of interest statement

The authors declare that the research was conducted in the absence of any commercial or financial relationships that could be construed as a potential conflict of interest.

## References

[B1] Agarwal S. M. Bose A. Shivakumar V. Narayanaswamy J. C. Chhabra H. Kalmady S. V.. (2015). Impact of antipsychotic medication on transcranial direct current stimulation (tDCS) effects in schizophrenia patients. Psychiatry Res. 235, 97–103. 10.1016/j.psychres.2015.11.042 26699879

[B2] Agras W. S. Lock J. Brandt H. Bryson S. W. Dodge E. Halmi K. A.. (2014). Comparison of 2 family therapies for adolescent anorexia nervosa: a randomized parallel trial. JAMA Psychiatry 71, 1279–1286. 10.1001/jamapsychiatry.2014.1025 25250660 PMC6169309

[B3] Aigner M. Treasure J. Kaye W. Kasper S. (2011). World Federation of Societies of Biological Psychiatry (WFSBP) guidelines for the pharmacological treatment of eating disorders. World J. Biol. Psychiatry 12, 400–443. 10.3109/15622975.2011.602720 21961502

[B4] Alcaro A. Huber R. Panksepp J. (2007). Behavioral functions of the mesolimbic dopaminergic system: an affective neuroethological perspective. Brain Res. Rev. 56, 283–321. 10.1016/j.brainresrev.2007.07.014 17905440 PMC2238694

[B5] Antal A. Alekseichuk I. Bikson M. Brockmöller J. Brunoni A. R. Chen R. (2017). Low intensity transcranial electric stimulation: safety, ethical, legal regulatory and application guidelines. Clin. Neurophysiol. 19:30212 10.1016/j.clinph.2017.06.001 PMC5985830 28709880

[B6] American Psychiatric Association (2006). Practice guideline for the treatment of patients with eating disorders (revision). Am. J. Psychiatry 163, 1–54. 10642782

[B6a] APA American Psychiatric Association (2013). Diagnostic and Statistical Manual of Mental Disorders, 5th edn. Arlington, VA: American Psychiatric Publishing.

[B7] Arcelus J. Mitchell A. J. Wales J. Nielsen S. (2011). Mortality rates in patients with anorexia nervosa and other eating disorders. A meta-analysis of 36 studies. Arch. Gen. Psychiatry 68, 724–731. 10.1001/archgenpsychiatry.2011.74 21727255

[B8] Avena N. M. Bocarsly M. E. (2012). Dysregulation of brain reward systems in eating disorders: neurochemical information from animal models of binge eating, bulimia nervosa, and anorexia nervosa. Neuropharmacology 63, 87–96. 10.1016/j.neuropharm.2011.11.010 22138162 PMC3366171

[B9] Bailer U. F. Frank G. K. Henry S. E. Price J. C. Meltzer C. C. Becker C.. (2007). Serotonin transporter binding after recovery from eating disorders. Psychopharmacology 195, 315–324. 10.1007/s00213-007-0896-7 17690869

[B10] Bär K. J. Berger S. Schwier C. Wutzler U. Beissner F. (2013). Insular dysfunction and descending pain inhibition in anorexia nervosa. Acta Psychiatr. Scand. 127, 269–278. 10.1111/j.1600-0447.2012.01896.x 22747702

[B11] Bardone-Cone A. M. Harney M. B. Maldonado C. R. Lawson M. A. Robinson D. P. Smith R.. (2010). Defining recovery from an eating disorder: conceptualization, validation, and examination of psychosocial functioning and psychiatric comorbidity. Behav. Res. Ther. 48, 194–202. 10.1016/j.brat.2009.11.001 19945094 PMC2829357

[B12] Bergen A. W. Yeager M. Welch R. A. Haque K. Ganjei J. K. van den Bree M. B.. (2005). Association of multiple DRD2 polymorphisms with anorexia nervosa. Neuropsychopharmacology 30, 1703–1710. 10.1038/sj.npp.1300719 15920508

[B13] Berridge K. Robinson T. (1998). What is the role of dopamine in reward: hedonic impact, reward learning, or incentive salience? Brain Res. 28, 309–369. 10.1016/S0165-0173(98)00019-8 9858756

[B14] Bodell L. P. Keel P. K. (2010). Current treatment for anorexia nervosa: efficacy, safety, and adherence. Psychol. Res. Behav. Manag. 3, 91–108. 10.2147/PRBM.S13814 22110333 PMC3218763

[B15] Boggiano M. M. Wenger L. E. Burgess E. E. Tatum M. M. Sylvester M. D. Morgan P. R.. (2017). Eating tasty foods to cope, enhance reward, socialize or conform: what other psychological characteristics describe each of these motives. J. Health Psychol. 22, 280–289. 10.1177/1359105315600240 26311817

[B16] Bohon C. Stice E. (2011). Reward abnormalities among women with full and subthreshold bulimia nervosa: a functional magnetic resonance imaging study. Int. J. Eat. Disord. 44, 585–595. 10.1002/eat.20869 21997421 PMC3111910

[B17] Brooks S. J. O'Daly OG. Uher R. Friederich H. C. Giampietro V. Brammer M.. (2011). Differential neural responses to food images in women with bulimia versus anorexia nervosa. PLoS ONE 6:22259. 10.1371/journal.pone.0022259 21799807 PMC3140495

[B18] Brunoni A. R. Amadera J. Berbel B. Volz M. S. Rizzerio B. G. Fregni F. (2011). A systematic review on reporting and assessment of adverse effects associated with transcranial direct current stimulation. Int. J. Neuropsychopharmacol. 14, 1133–1145. 10.1017/S1461145710001690 21320389

[B19] Bulik CM. Sullivan P. F. Fear J. Pickering A. (1997). Predictors of the development of bulimia nervosa in women with anorexia nervosa. J. Nerv. Ment. Dis. 185, 704–707. 10.1097/00005053-199711000-00009 9368548

[B20] Casper R. C. (2006). The “drive for activity” and “restlessness” in anorexia nervosa: potential pathways. J. Affect. Disord. 92, 99–107. 10.1016/j.jad.2005.12.039 16448703

[B20a] Celone K. A. Thompson-Brenner H. Ross R. S. Pratt E. M. Stern C. E. (2011). An fMRI investigation of the fronto-striatal learning system in women who Exhibit eating disorder behaviors. Neuroimage 56, 1749–1757. 10.1016/j.neuroimage.2011.03.02 21419229 PMC3860103

[B21] Cuzzolaro M. Vetrone G. Marano G. Garfinkel P. E. (2006). The Body Uneasiness Test (BUT): development and validation of a new body image assessment scale. Eat. Weight Disord. 11, 1–13. 10.1007/BF03327738 16801740

[B22] Diana M. (2011). The dopamine hypothesis of drug addiction and its potential therapeutic value. Front. Psychiatry 2:64. 10.3389/fpsyt.2011.00064 22144966 PMC3225760

[B23] Doherty J. M. Schier C. J. Vena A. A. Geoffrey A. D. Rueben A. G. (2016). Medial prefrontal cortical dopamine responses during operant self-administration of sweetened ethanol. Alcohol. Clin. Exp. Res. 40, 1662–1670. 10.1111/acer.13141 27435872 PMC5573256

[B24] Dold M. Aigner M. Klabunde M. Treasure J. Kasper S. (2015). Second-generation antipsychotic drugs in anorexia nervosa: a meta-analysis of randomized controlled trials. Psychother. Psychosom. 21, 110–116. 10.1159/000369978 25722106

[B25] Ehrlich S. Geisler D. Ritschel F. King J. A. Seidel M. Boehm I.. (2015). Elevated cognitive control over reward processing in recovered female patients with anorexia nervosa. J. Psychiatry Neurosci. 40, 307–315. 10.1503/jpn.140249 26107161 PMC4543093

[B26] Fairburn C. G. Harrison P. J. (2003). Eating disorders. Lancet 361, 407–416. 10.1016/S0140-6736(03)12378-1 12573387

[B26a] Fisher W. W. Piazza C. C. Bowman L. G. Kurtz P. F. Sherer M. R. Lachman S. R. (1994). A preliminary evaluation of empirically derived consequences for the treatment of pica. J. Appl. Behav. Anal. 27, 447–457. 10.1901/jaba.1994.27-447 7928789 PMC1297826

[B27] Fonteneau C. Redoute J. Haesebaert F. Le Bars D. Costes N. Suaud-Chagny M. F.. (2018). Frontal transcranial direct current stimulation induces dopamine release in the ventral striatum in human. Cereb. Cortex 28, 2636–2646. 10.1093/cercor/bhy093 29688276 PMC5998959

[B28] Frank G. K. Shott M. E. Hagman J. O. Schiel M. A. DeGuzman M. C. Rossi B. (2017). The partial dopamine D2 receptor agonist aripiprazole is associated with weight gain in adolescent anorexia nervosa. Int. J. Eat. Disord. 50, 447–450. 10.1002/eat.22704 28334444 PMC5392387

[B29] Fregni F. Orsati F. Pedrosa W. Fecteau S. Tome F. A. Nitsche M. A.. (2008). Transcranial direct current stimulation of the prefrontal cortex modulates the desire for specific foods. Appetite 51, 34–41. 10.1016/j.appet.2007.09.016 18243412 PMC3541023

[B30] Fresnoza S. Stiksrud E. Klinker F. Liebetanz D. Paulus W. Kuo M. F. (2014). Dosage-dependent effect of dopamine D2 receptor activation on motor cortex plasticity in humans. J. Neurosci. 6, 10701–10709. 10.1523/JNEUROSCI.0832-14.2014 PMC4122803 25100602

[B31] Friederich H. C. Herzog W. (2011). Cognitive-behavioral flexibility in anorexia nervosa. Curr. Top. Behav. Neurosci. 6, 111–123. 10.1007/7854_2010_83 21243473

[B32] Galusca B. Costes N. Zito N. G. Peyron R. Bossu C. Lang F.. (2008). Organic background of restrictive-type anorexia nervosa suggested by increased serotonin 1A receptor binding in right frontotemporal cortex of both lean and recovered patients. Biol. Psychiatry 64, 1009–1013. 10.1016/j.biopsych.2008.06.006 18639866

[B33] Garner D. M. (2004). Eating Disorder Inventory-3. Professional Manual. Lutz, FL: Psychological Assessment Resources.

[B34] Garner D. M. Olmsted M. P. Bohr Y. Garfinkel P. E. (1982). The eating attitudes test: psychometric features and clinical correlates. Psychol. Med. 12, 871–878. 10.1017/S0033291700049163 6961471

[B35] Garner D. Desmond M. Desai J. Lockert J. (2016). The disconnect between treatment outcome data and reimbursement for the treatment of anorexia nervosa. Int J. Physiatry 2, 1–6. 10.23937/2572-4215.1510006

[B36] Giannini M. Pannocchia L. Dalle Grave R. Muratori F. (2008). Adattamento Italiano dell'EDI-3. Eating Disorder Inventory-3. Firenze: Giunti O.S. Organizzazioni Speciali.

[B37] Gillman M. A. Lichtigfeld F. J. (1986). The opioids, dopamine, cholecystokinin, and eating disorders. Clin. Neuropharmacol. 9, 91–97. 10.1097/00002826-198602000-00011 3548955

[B38] Goldman R. L. Borckardt J. J. Frohman H. A. O'Neil P. M. Madan A. Campbell L. K.. (2011). Prefrontal cortex transcranial direct current stimulation (tDCS) temporarily reduces food cravings and increases the self-reported ability to resist food in adults with frequent food craving. Appetite 56, 741–746. 10.1016/j.appet.2011.02.013 21352881

[B39] Grace A. A. (1991). Phasic versus tonic dopamine release and the modulation of dopamine system responsivity: a hypothesis for the etiology of schizophrenia. Neuroscience 41, 1–24. 10.1016/0306-4522(91)90196-U 1676137

[B40] Grunwald M. Weiss T. Assmann B. Ettrich C. (2004). Stable asymmetric interhemispheric theta power in patients with anorexia nervosa during haptic perception even after weight gain: a longitudinal study. J. Clin. Exp. Neuropsychol. 26, 608–620. 10.1080/13803390409609785 15370383

[B41] Guarda A. (2008). Treatment of anorexia nervosa: insights and obstacles. Physiol. Behav. 94, 113–120. 10.1016/j.physbeh.2007.11.020 18155737

[B42] Gustavsson A. Svensson M. Jacobi F. Allgulander C. Alonso J. Beghi E.. (2011). Cost of disorders of the brain in Europe 2010. Eur. Neuropsychopharmacol. 21, 718–779. 10.1016/j.euroneuro.2011.08.008 21924589

[B43] Halmi K. (1982). Pragmatic information on the eating disorders. Psychiatr. Clin. North Am. 5, 371–377. 10.1016/S0193-953X(18)30873-6 6750574

[B44] Hamamura T. Harada T. (2007). Unique pharmacological profile of aripiprazole as the phasic component buster. Psychopharmacology 191, 741–743. 10.1007/s00213-006-0654-2 17205315

[B45] Hare T. A. Camerer C. F. Rangel A. (2009). Selfcontrol in decision-making involves modulation of the vmPFC valuation system. Science 324, 646–648. 10.1126/science.1168450 19407204

[B46] Herpertz-Dahlmann B. de Zwaan M. (2011). Eating disorders: new findings for diagnosis and treatment. Nervenarzt 82, 1091–1092. 10.1007/s00115-010-3226-y 21853376

[B47] Hestad K. A. Weider S. Nilsen K. B. Indredavik M. S. Sand T. (2016). Increased frontal electroencephalogram theta amplitude in patients with anorexia nervosa compared to healthy controls. Neuropsychiatr. Dis. Treat. 21, 2419–2423. 10.2147/NDT.S113586 PMC5036600 27703359

[B47a] Isern R. D. (1987). Family violence and the Klüver-Bucy syndrome. South Med. J. 80, 373–377. 3824025 10.1097/00007611-198703000-00026

[B48] Kamolz S. Richter M. M. Schmidtke A. Fallgatter A. J. (2008). Transcranial magnetic stimulation for comorbid depression in anorexia. Nervenarzt 79, 1071–1073. 10.1007/s00115-008-2537-8 18661116

[B49] Kaye W. H. Fudge J. L. Paulus M. (2009). New insights into symptoms and neurocircuit function of anorexia nervosa. Nat. Rev. Neurosci. 10, 573–584 10.1038/nrn2682 19603056 PMC13038070

[B50] Kaye W. H. Wagner A. Fudge J. L. Paulus M. (2010). Neurocircuity of eating disorders. Curr. Top. Behav. Neurosci. 6, 37–57. 10.1007/7854_2010_85 21243469 PMC5957512

[B51] Kaye W. H. Wierenga C. E. Bailer U. F. Simmons A. N. Bischoff-Grethe A. (2013). Nothing tastes as good as skinny feels: the neurobiology of anorexia nervosa. Trends Neurosci. 36, 110–120. 10.1016/j.tins.2013.01.003 23333342 PMC3880159

[B52] Khedr E. M. Elfetoh N. A. Ali A. M. Noamany M. (2014). Anodal transcranial direct current stimulation over the dorsolateral prefrontal cortex improves anorexia nervosa: a pilot study. Restor. Neurol. Neurosci. 32, 789–797. 10.3233/RNN-140392 25189181

[B53] Klauss J. Penido Pinheiro L. C. Silva Merlo B. L. de Almeida Correia Santos G. Fregni F. Nitsche M. A.. (2014). A randomized controlled trial of targeted prefrontal cortex modulation with tDCS in patients with alcohol dependence. Int. J. Neuropsychopharmacol. 17, 1793–1803. 10.1017/S1461145714000984 25008145

[B54] Kontis D. Theochari E. (2012). Dopamine in anorexia nervosa: a systematic review. Behav. Pharmacol. 23, 496–515. 10.1097/FBP.0b013e328357e115 22854306

[B55] Kovacs M. (1982). C.D.I.: Children's Depression Inventory. Questionario di Autovalutazione. Adattamento Italiano a Cura di Camuffo M., Cerutti R., Lucarelli L., Mayer R. (1988). Firenze: Organizzazioni Speciali.

[B56] Kovacs M. (1992). Children's Depression Inventory (CDI) Manual. New York, NY: Multi Health Systems.

[B57] Kullmann S. Giel K. E. Hu X. Bischoff S. C. Teufel M. Thiel A.. (2014). Impaired inhibitory control in anorexia nervosa elicited by physical activity stimuli. Soc. Cogn. Affect. Neurosci. 9, 917–923. 10.1093/scan/nst070 23677490 PMC4090959

[B58] Laghi F. Pompili S. Zanna V. Castiglioni M. C. Criscuolo M. Chianello I.. (2017). How adolescents with anorexia nervosa and their parents perceive family functioning? J. Health Psychol. 22, 197–207. 10.1177/1359105315597055 26253650

[B59] Le Grange D. Lock J. Agras W. S. Moye A. Bryson S. W. Jo B.. (2012). Moderators and mediators of remission in family-based treatment and adolescent focused therapy for anorexia nervosa. Behav. Res. Ther. 50, 85–92. 10.1016/j.brat.2011.11.003 22172564 PMC3260378

[B60] Lee D. J. Elias G. J. B. Lozano A. M. (2018). Neuromodulation for the treatment of eating disorders and obesity. Ther. Adv. Psychopharmacol. 8, 73–92. 10.1177/2045125317743435 29399320 PMC5788100

[B61] Ljubisavljevic M. Maxood K. Bjekic J. Oommen J. Nagelkerke N. (2016). Long-term effects of repeated prefrontal cortex Transcranial Direct Current Stimulation (tDCS) on food craving in normal and overweight young adults. Brain Stimul. 9, 826–833. 10.1016/j.brs.2016.07.002 27498606

[B62] Lock J. Le Grange D. Agras W. S. Moye A. Bryson S. W. Jo B. (2010). Randomized clinical trial comparing family-based treatment with adolescent-focused individual therapy for adolescents with anorexia nervosa. Arch. Gen. Psychiatry 67, 1025–1032. 10.1001/archgenpsychiatry.2010.128 20921118 PMC3038846

[B63] Lowe C. J. Hall P. A. Staines W. R. (2014). The effects of continuous theta burst stimulation to the left dorsolateral prefrontal cortex on executive function, food cravings, and snack food consumption. Psychosom. Med. 76, 503–511. 10.1097/PSY.0000000000000090 25215552

[B64] March J. S. Parker J. D. Sullivan K. Stallings P. Conners C. K. (1997). The Multidimensional Anxiety Scale for Children (MASC): factor structure, reliability, and validity. J. Am. Acad. Child Adolesc. Psychiatry 36, 554–565. 10.1097/00004583-199704000-00019 9100431

[B65] McClelland J. Bozhilova N. Campbell L. Schmidt U. (2013). A Systematic review of the effects of neuromodulation on eating and body weight: evidence from human and animal studies. Eur. Eat. Disord. Rev. 21, 436–455. 10.1002/erv.2256 24155246

[B66] McClelland J. Kekic M. Bozhilova N. Nestler S. Dew T. Van den Eynde F.. (2016a). A randomised controlled trial of neuronavigated repetitive transcranial magnetic stimulation (rTMS) in anorexia nervosa. PLoS ONE 11:148606. 10.1371/journal.pone.0148606 27008620 PMC4805273

[B67] McClelland J. Kekic M. Campbell I. C. Schmidt U. (2016b). Repetitive transcranial magnetic stimulation (rTMS) treatment in enduring anorexia nervosa: a case series. Eur. Eat. Disord. Rev. 24, 157–163. 10.1002/erv.2414 26537308

[B68] Miller E. K. Cohen J. D. (2001). An integrative theory of prefrontal cortex function. Annu. Rev. Neurosci. 24, 167–202. 10.1146/annurev.neuro.24.1.167 11283309

[B69] Mohr H. M. Zimmermann J. Röder C. Lenz C. Overbeck G. Grabhorn R. (2010). Separating two components of body image in anorexia nervosa using fMRI. Psychol. Med. 40, 1519–1529. 10.1017/S0033291709991826 19917143

[B70] Montenegro R. A. Okano A. H. Cunha F. A. Gurgel J. L. Fontes E. B. Farinatti P. T. (2012). Prefrontal cortex transcranial direct current stimulation associated with aerobic exercise change aspects of appetite sensation in overweight adults. Appetite 58, 333–338. 10.1016/j.appet.2011.11.008 22108669

[B71] Monte-Silva K. Kuo M. F. Thirugnanasambandam N. Liebetanz D. Paulus W. Nitsche M. A. (2009). Dose-dependent inverted U-shaped effect of dopamine (D2-like) receptor activation on focal and nonfocal plasticity in humans. J. Neurosci. 29, 6124–6131. 10.1523/JNEUROSCI.0728-09.2009 19439590 PMC6665507

[B72] Nasseri P. Nitsche M. A. Ekhtiari H. (2015). A framework for categorizing electrode montages in transcranial direct current stimulation. Front. Hum. Neurosci. 9:54. 10.3389/fnhum.2015.00054 25705188 PMC4319395

[B73] National Institute for Clinical Excellence (2004). National Collaborating Centre for Mental Healt, Eating Disorders. (NICE) Core Interventions in the Treatment and Management of Anorexia Nervosa, Bulimia Nervosa, and Related Eating Disorders. Leicester, UK: British Psychological Society. 23346610

[B74] Nitsche M. A. Paulus W. (2000). Excitability changes induced in the human motor cortex by weak transcranial direct current stimulation. J. Physiol. 527(Pt 3), 633–639. 10.1111/j.1469-7793.2000.t01-1-00633.x 10990547 PMC2270099

[B75] Nitsche M. A. Boggio P. S. Fregni F. Pascual-Leone A. (2009). Treatment of depression with transcranial direct current stimulation (tDCS): a review. Exp. Neurol. 219, 14–19. 10.1016/j.expneurol.2009.03.038 19348793

[B76] Nitsche M. A. Lampe C. Antal A. Liebetanz D. Lang N. Tergau F.. (2006). Dopaminergic modulation of long-lasting direct current-induced cortical excitability changes in the human motor cortex. Eur. J. Neurosci. 23, 1651–1657. 10.1111/j.1460-9568.2006.04676.x 16553629

[B77] Nitsche M. A. Liebetanz D. Antal A. Lang N. Tergau F. Paulus W. (2003). Modulation of cortical excitability by weak direct current stimulation – technical, safety and functional aspects. Suppl. Clin. Neurophysiol. 56, 255–276. 10.1016/S1567-424X(09)70230-2 14677403

[B78] Nitsche M. A. Muller-Dahlhaus F. Paulus W. Ziemann U. (2012). The pharmacology of neuroplasticity induced by non-invasive brain stimulation: building models for the clinical use of CNS active drugs. J. Physiol. 590, 4641–4662. 10.1113/jphysiol.2012.232975 22869014 PMC3487028

[B79] Normann C. Schmitz D. Fürmaier A. Döing C. Bach M. (2007). Long-term plasticity of visually evoked potentials in humans is altered in major depression. Biol. Psychiatry 62, 373–380. 10.1016/j.biopsych.2006.10.006 17240361

[B80] Oberndorfer T. Simmons A. McCurdy D. Strigo I. Matthews S. Yang T.. (2013). Greater anterior insula activation during anticipation of food images in women recovered from anorexia nervosa versus controls. Psychiatry Res. 214, 132–141. 10.1016/j.pscychresns.2013.06.010 23993362 PMC3880160

[B81] Owesson-White C. A. Roitman M. F. Sombers L. A. Belle A. M. Keithley R. B. Peele J. L.. (2012). Sources contributing to the average extracellular concentration of dopamine in the nucleus accumbens. J. Neurochem. 121, 252–262. 10.1111/j.1471-4159.2012.07677.x 22296263 PMC3323736

[B82] Phillipou A. Gurvich C. Castle D. J. Abel L. A. Rossell S. L. (2015). Comprehensive neurocognitive assessment of patients with anorexia nervosa. World J. Psychiatry 22, 404–411. 10.5498/wjp.v5.i4.404 PMC4694554 26740932

[B83] Raven J. C. (1994). CPM, Coloured Progressive Matrices. Firenze: Giunti OrganizzazioniSpeciali. (Italian Adaptation, 2008).

[B84] Ridding M. C. Ziemann U. (2010). Determinants of the induction of cortical plasticity by non-invasive brain stimulation in healthy subjects. J. Physiol. 588(Pt 13) 2291–2304. 10.1113/jphysiol.2010.190314 20478978 PMC2915507

[B85] Rienecke R. D. (2017). Family-based treatment of eating disorders in adolescents: current insights. Adolesc. Health Med. Ther. 1, 69–79. 10.2147/AHMT.S115775 PMC5459462 28615982

[B86] Ritschel F. Geisler D. King J. A. Bernardoni F. Seidel M. Boehm I.. (2017). Neural correlates of altered feedback learning in women recovered from anorexia nervosa. Sci. Rep. 7:5421. 10.1038/s41598-017-04761-y 28710363 PMC5511172

[B87] Riva G. (2016). Neurobiology of anorexia nervosa: serotonin dysfunctions link self-starvation with body image disturbances through an impaired body memory. Hum Neurosci. 24:10 10.3389/fnhum.2016.00600 PMC5121233 27932968

[B88] Smink F. R. van Hoeken D. Hoek H. W. (2012). Epidemiology of eating disorders: incidence, prevalence and mortality rates. Curr. Psychiatry Rep. 14, 406–414. 10.1007/s11920-012-0282-y 22644309 PMC3409365

[B89] Södersten P. Bergh C. Leon M. (2016). Commentary: new insights in anorexia nervosa. Front. Neurosci. 10:483. 10.3389/fnins.2016.00483 27826227 PMC5078500

[B90] Steinglass J. E. Lempert K. M. Choo T. H. Kimeldorf M. B. Wall M. Walsh B. T.. (2017). Temporal discounting across three psychiatric disorders: anorexia nervosa, obsessive compulsive disorder, and social anxiety disorder. Depress. Anxiety 34, 463–470. 10.1002/da.22586 28009473 PMC5869031

[B91] Steinglass J. Walsh B. T. (2006). Habit learning and anorexia nervosa: a cognitive neuroscience hypothesis. Int. J. Eat. Disord. 39, 267–275. 10.1002/eat.20244 16523472

[B92] Steinhausen H. C. (2002). The outcome of anorexia nervosa in the 20th century. Am. J. Psychiatry 159, 1284–1293. 10.1176/appi.ajp.159.8.1284 12153817

[B93] Striegel-Moore R. H. Franko D. L. Thompson D. Barton B. Schreiber G. B. Daniels S. R. (2004). Changes in weight and body image over time in women with eating disorders. Int. J. Eat. Disord. 36, 315–327. 10.1002/eat.20053 15478134

[B94] Strigo I. A. Matthews S. C. Simmons A. N. Oberndorfer T. Klabunde M. Reinhardt L. E.. (2013). Altered insula activation during pain anticipation in individuals recovered from anorexia nervosa: evidence of interoceptive dysregulation. Int. J. Eat. Disord. 46, 23–33. 10.1002/eat.22045 22836447 PMC3507323

[B95] Tanaka T. Takano Y. Tanaka S. Hironaka N. Kobayashi K. Hanakawa T.. (2013). Transcranial direct-current stimulation increases extracellular dopamine levels in the rat striatum. Front. Syst. Neurosci. 7:6. 10.3389/fnsys.2013.00006 23596399 PMC3622879

[B96] Van den Eynde F. Guillaume S. Broadbent H. Campbell I. C. Schmidt U. (2013). Repetitive transcranial magnetic stimulation in anorexia nervosa: a pilot study. Eur. Psychiatry 28, 98–101. 10.1016/j.eurpsy.2011.06.002 21880470

[B98] Van Kuyck K. Gérard N. Van Laere K. Casteels C. Pieters G. Gabriëls L.. (2009). Towards a neurocircuitry in anorexia nervosa: evidence from functional neuroimaging studies. J. Psychiatr. Res. 43, 1133–1145. 10.1016/j.jpsychires.2009.04.005 19442986

[B99] Vierheilig N. Mühlberger A. Polak T. Herrmann M. J. (2016). Transcranial direct current stimulation of the prefrontal cortex increases attention to visual target stimuli. J. Neural. Transm. 123, 1195–1203. 10.1007/s00702-016-1542-5 27059880

[B100] Wagner A. Aizenstein H. Venkatraman V. K. Bischoff-Grethe A. Fudge J. May J. C.. (2010). Altered striatal response to reward in bulimia nervosa after recovery. Int. J. Eat. Disord. 43, 289–294. 10.1002/eat.20699 19434606 PMC4286149

[B101] Walsh B. T. (2013). The enigmatic persistence of anorexia nervosa. Am. J. Psychiatry 170, 477–484. 10.1176/appi.ajp.2012.12081074 23429750 PMC4095887

[B102] Wietschorke K. Lippold J. Jacob C. Polak T. Herrmann M. J. (2016). Transcranial direct current stimulation of the prefrontal cortex reduces cue-reactivity in alcohol-dependent patients. J. Neural Transm. 123, 1173–1178. 10.1007/s00702-016-1541-6 27038632

[B103] Ziadi Trives M. Bonete Llácer J. M. García Escudero M. A. Martínez Pastor C. J. (2013). Effect of the addition of aripiprazole on hyperprolactinemia associated with risperidone long-acting injection. J. Clin. Psychopharmacol. 33, 538–541. 10.1097/JCP.0b013e3182970431 23775053

